# 

**Published:** 1846-06-01

**Authors:** 


### INDEX TO VOLUME SECOND.

Anasarca and Ascites, cases of by G. N. Burwell, M. D. 1
Acton on Venereal Diseases, 122
Anatomical Museum, 427
Anatomical Materiel, 534
Academy of M. and S. in New-York, 560
Associations Medical, voluntary, 630
Adipocere, case of, 671
Atlee, Prof. valedictory, 748
Bartlett on Philosophy of Medical Science, review of, 11
Boston Society Med. improvement, 112
Brodie on Surgery, 122
Brodie, Clinical Lectures, 145
Brights disease, Burwell on, 125, 185, 340
Burwell, case of Diabetes Mellitus. 389
Burwell, on Morbid conditions of blood 310
Blood, method of analyses of, 170
Bowditch's Young Stethoscopist, review of, 225
Buffalo, health of, 246
Bigelow's Orthopedic Surgery, 250
Blood, Physiology and Pathology of, 283, 552
Beck on Emetics in Young subjects, 289
Barbarism in N. Y. 313
Bell and Stokes' Practice, 372
Buffalo City Hospital, 376, 436
Blood letting, remarks on, by Editor, 377, 450
Bladder, rupture of, case, 419
Bartlett, Prof., letter from, 497
Beck on adulteration of Medicines, 501
Beck on Mercury in Young subjects, 542
Bell Dr., valedictory, 559
Bedford's, Prof., Introductory, 565
Bennett on the Uterus, review, 575
Carotid Arteries, ligature of, by J. Mason Warren, 41
Convention, National Medical, 51, 682, 313, 499, 432
Convention, Medical in Ill. and Wis. 56
Compensation for Public services of Medical men 57
Chapman's compendium of Lectures, 122
Conger, case of Enterotomy, 133
Chalybeate water, extemporaneous, 171
Carpenter's Physiology, review of, 215
Cutler's Anatomy and Physiology, 251
Coley on diseases of Children, 252
Coventry on Proceedings of N. Y. State Medical Society, 291
Croup, surgical treatment of, 301
Creosote in Diarrhœa, 360
Corrigan on stimulants in Fever, 421
Chemistry of the four seasons, 437
Coxe's Epitome of Hippocrates and Galen, 438
Conjunctivitis catarrhal, 484
Corrosive sub.. incompatibles with, 490
Conium, inspissated extract of, 562
Clymer on Fevers, 565
Congar's Meteorological and Path. Report. 600
Croup, obs. on, by Prof. Stevens, 611
Cliniques, 623
Circular State Med. Society, 626
Croup treated by cauterizing larynx 728
Delusion Medical, force of, 59
Delirium Tremens, by Lieut. Long, M. D. 107
Dowler on Post Mortem contractility, 152
Deaths in London, 315
Dental Science, Journal and Library of, 373
Delirium Tremens, by Dr. Corrigan, 424
Diarrhœa chronic, 487
Draper, Prof., on circulation, 521
Dropsy, Linton's recipes for, 525
Desmarres, Prof., 534
Darracks, Prof.. Introductory lecture, 565
Dislocation of five months standing reduced, 731
Diabetes, Sugar in expectoration, 733
Doctors and Lawyers, summary way of making, 741
Epilepsy cured by ligature of carotids, 119
Erie Co. Med. Society, resolutions of, 120
Erysipelas, treatment of, by Velpeau, 423
Ether, vapor inhalation of, 433, 469, 491 549, 674, 691, 743, 750
Ethics Medical, 536
Excito-motary system, anatomy of, 539
Fordyce on Fever, notice of, 61
Fees from a Pasha, 428
Fever Intermittent, treatment of, 429
Fox on teeth, 460
Fevers, remittent and typhoid at Buffalo, 554
Frank W., *vs*. State of New York, 557
Fever, identity of typhus and typhoid, 558
Fever miasmatic, statistics of, 666
Fevers, treatment without mercury, 675
Fever typhoid, treatment of, 735
Foecal accumulation in rectum, 737
Gross' Path. Anatomy, notice of, 368
Gerhard on chest, 373
Gardener John, address by, 409
Griffin on diagnosis abdom. affections, 416
Gangrene, new remedy for, 533
Homœopathy, report on by Dr. Hoyt, 52
Hughes on physical diagnosis, notice of, 61
Hamilton's European Tour, 65, 139, 198, 257, 396, 631, 690
Hyde, cases of dislocation of shoulder, 81
Hyde, report of cases, 455
Homœopathy, &c., by John Forbes, 92
Homœopathy, 122, 311, 526, 312
Hoblin's chemistry, 122
Hamilton, Prof., on fracture of skull, 347
Hamilton, Prof., surgical clinique, 303 567, 637
Hamilton, Prof., on new instrument for extension in dislocation of small joints, 645
Hasse's path. anatomy, 499
Horner's dissector, 500
House, Dr. J. G. on epidemic hepititis, 619
Hepatitis epidemic, 620
Hydro cephalus, case of, 619

Hall C. A. Dr. case of poisoning by arsenic, 643
House, Dr. C., case of carcinoma of stomach, 718
Johnson on trop. climates, notice of 61
Inhumation of living persons, 315
Insanity homicidal, case of, 524
Idiot's asylum for, report, 690
Insanity, treatment of, by Dr. Birgham, 731
Ischuria renalis, case of, 739
Kermes mineral in diseases of respiratory organs, 168
Liston's surgery, with Mutter's notes, notice of, 61, 261, 331
Liston's Surgery. by Gross, 502
Latham on diseases of Heart, 654
Leucorrhea, chloride of lime in, 743
Mott's Velpeau's op. Surg., reviews of, 23, 83. 710
Medical books in Buffalo, 56
Medical Colleges, statistics of, 121
Mortality, comparative, of Am. cities, 179
Medical prescriptions, errors in translating, 182
Mal practice, suit for, 183, 311, 364
Medical school at Buffalo, 244
Medical instruction private, 245
Mercer, Dr. letter from, 304
Mass. Med. College, circular of, 311
Manslaughter, indictment for, 315
Medical profession, address to, by Com. appointed by Nat. Med. Con. 365
Medical periodical literature, 374
Medical Dept. Univ. of Buffalo, 435, 566
M'Neven, case of abnormal cyst in parturition, 527
Medical College at Philadelphia, 561
Medical reform, new plan of, 616
Mitchell, Dr. cases of Fever. 646
Medical Profession, 649
Matico, properties of, 681
Medical College Dispensary, cases at, 699
Mott's Clinique, 729
Nostrum certifiers, 312
Nœvus Maternus, 486
New-York Med. Society, charter of, 489
New Year's Day, 494
Neil on Arteries and Nerves, 500
New-York Jour. of Med., Editor of, 692
Originality, questions of, settled, 52
Ova periodical maturation and discharge of, 112
Puerperal Fever, connection of, with Epidemic Erysipelas, 48
Pinel, celebration of birth day of, at Utica Asylum, 60
Prescriptions, Latin, 60
Poirrgo, lotions in, 171
Physical diagnosis without a master, 172
Pneumonitis Lobular, 242
Phillips on Scrofula, review of, 251, 270, 317
Physiological Practitioner, 361
Purple on Corpus Luteum, 412
Parker's, Prof., Clinique, 483
Pregnancy and delivery during prolapsus, 533
Proceedings Society State of N. Y. 692
Professors, election of by concours, 744
Pericardii Hydrops suddenly formed, 746
Quarantine Laws, report on, by com. of House of Assembly State of N.Y. 100
Quinine, endemic application of, 240
Quinine, large doses in Typhoid Fever, 241
Quinine in Acute Rheumatism, 242
Quinine, cause of blindness, Professor M'Lean, 530
Quintard on poultices to inflamed eyes, 529
Quack Doctors and Medicines, 534
Reform Medical, Resolutions N. Y. Med. Soeiety. 58
Remittent Fever, path. characters of, 235
Remittent Fever, cold affusions in, 299
Remittent Fever in Buffalo, 555
Remittent Fever, thoughts on, 622
Royle's Materia Medica, 563
Reid, Dr. W., case Hæma temesis, 641
State Med. Society, officers of, 60
Sprague, Dr. A. S., case of extra uterine fœtation, 68
Strangury, new remedy for, 169
Syphilis constitutional, case of, 414
Sutton on Scrofula, 415
Smith's minor Surgery, 439
Sex doubtful, case of, 535
Skeleton to, poetical contribution, 687
Small Pox, ectrotic treatment of, 725
Sounds from corpse, 742
Transactions Med. Society, State of N. York, Vol. VI, Part III. 32
Transactions of Med. College of Physicians of Philadelphia, 41, 462
Trismus Nascentium, pathology of, by Sims, 39
Tamlin on deformities, notice of, 61
Tendons, section and recuperative property of, 137
Toothache, remedy for, 525
Urine, clinical chem., introduction to, 110
Univ. of N.Y. annual announcement, 115
Uterus, spontaneous rupture of, 4th m. 214
Urinary changes, diagnosis of, by Ed. 441
Urinometer, remarks on, 548
Velpeau on fractures, 363
Vexations, 433
Vethake's Encyclopœdia, 562
Vagina double, cases of, 679
Warren on Physical Education, 251
Water Cure, 315
Wollaston, 327
White, Prof. case occlusion of vagina and os uteri, 391
Williams' Med. Biography, 501
Water Cure by John Forbes, review, 551
Westcott's Prof. introductory, 566
Watson, Dr. on Practical Education, 597
Wounds from fire arms without ball, 668
Warren, Prof. resignation of, 691

☞ At the last Annual Meeting of the N. Y. State Medical Society, held at Albany, in February, 1846, the following gentlemen were elected officers for the ensuing year, viz :

*President*, JOHN McCALL, M. D., of Utica.
*Vice President*, STEPHEN HASBROUCK, M. D., of New-York,
*Secretary*, PETER VAN BUREN, M. D., of Albany,
*Treasurer*, PETER VAN OLINDA, M. D., of Albany.

